# Endotoxin-Binding Peptides Derived from Casein Glycomacropeptide Inhibit Lipopolysaccharide-Stimulated Inflammatory Responses via Blockade of NF-κB activation in macrophages

**DOI:** 10.3390/nu7053119

**Published:** 2015-04-27

**Authors:** Xue Cheng, Dongxiao Gao, Bin Chen, Xueying Mao

**Affiliations:** 1 Key Laboratory of Functional Dairy, College of Food Science and Nutritional Engineering, China Agriculture University, Beijing 100083, China; E-Mails: xuecheng@163.com (X.C.); dongxiao@163.com (D.G.); 2 Key Laboratory of Space Nutrition and Food Engineering, China Astronaut Training Center, Beijing 100094, China; 3 Synergetic Innovation Center of Food Safety and Nutrition, Northeast Agriculture University, Haerbin 150030, China; E-Mail: chenb12@aliyun.com

**Keywords:** casein glycomacropeptide, LPS-binding, inflammation, toll-like receptor 4, nuclear factor-κB

## Abstract

Systemic low-grade inflammation and increased circulating lipopolysaccharide (LPS) contribute to metabolic dysfunction. The inhibitory effects and underlying molecular mechanisms of casein glycomacropeptide (GMP) hydrolysate on the inflammatory response of LPS-stimulated macrophages were investigated. Results showed that the inhibitory effect of GMP hydrolysates obtained with papain on nitric oxide (NO) production were obviously higher than that of GMP hydrolysates obtained with pepsin, alcalase and trypsin (*p* < 0.05), and the hydrolysate obtained with papain for 1 h hydrolysis (GHP) exhibited the highest inhibitory effect. Compared with native GMP, GHP markedly inhibited LPS-induced NO production in a dose-dependent manner with decreased mRNA level of inducible nitric oxide synthase (iNOS). GHP blocked toll-like receptor 4 (TLR4)/myeloid differentiation primary response 88 (MyD88)/nuclear factor-κB (NF-κB) signaling pathway activation, accompanied by downregulation of LPS-triggered significant upregulation of tumor necrosis factor (TNF)-α and interleukin (IL)-1β gene expression. Furthermore, GHP could neutralize LPS not only by direct binding to LPS, but also by inhibiting the engagement of LPS with the TLR4/MD2 complex, making it a potential LPS inhibitor. In conclusion, these findings suggest that GHP negatively regulates TLR4-mediated inflammatory response in LPS-stimulated RAW264.7 cells, and therefore may hold potential to ameliorate inflammation-related issues.

## 1. Introduction

Low-grade systemic inflammation is considered a hallmark of several metabolic diseases, including obesity, non-alcoholic fatty liver disease (NAFLD) and type 2 diabetes mellitus (T2DM) [1,2,3]. Recent insight suggests that an altered composition and diversity of gut microbiota plays an important role in the development of this low-grade inflammatory state. It has been found that excess fat-enriched diet consumption produced an increase in the Gram-negative/Gram-positive ratio of gut microbiota, which results in elevated plasma bacterial endotoxin (also known as lipopolysaccharide (LPS) concentrations [4]. Continuous subcutaneous LPS infusion, which was coupled with increased expression of toll-like receptor (TLR) 4 and nuclear factor-κB (NF-κB) in mononuclear cells, resulted in elevated secretion of pro-inflammatory mediators, such as nitric oxide (NO), tumor necrosis factor (TNF)-α and interleukin (IL)-1β [5,6]. These chemicals, in turn, serve as endogenous mediators of inflammation to trigger insulin resistance, which will favor hyperlipidemia [7]. Macrophages, which can enter target tissues and gain the functional attributes of their residence, are a cell type that is differentiated from monocytes in response to various inflammatory stimuli [8]. Previous studies have shown that almost all TNF-α and significant amounts of inducible nitric oxide synthase (iNOS) existing in adipose tissue were secreted by adipose tissue macrophages in obese subjects [9,10]. Macrophage-specific gene KO animals display dramatically reduced inflammatory pathway gene expression with decreased tissue cytokine levels in adipose tissue and liver [11]. Consequently, additional supplementation of LPS-binding and/or anti-inflammatory agents may have protective effects on metabolic diseases. For example, salsalate, a nonsteroidal anti-inflammatory drug, could make significant improvements in glycemia and adiponectin in obese individuals [12]. Polymyxin B (PMB), which can specifically eliminate Gram-negative bacteria and further bind LPS, could diminish hepatic steatosis [13]. However, their side effects, such as neurotoxicity and gastrointestinal adverse effects, may limit their wide use [14,15]. Natural and safe constituents from food have traditionally been and continue to be used for the treatment of metabolic syndrome. *Pinus densiflora* Sieb. et Zucc. (PSZ) effectively reversed NAFLD symptoms by decreasing lipid accumulation, inflammatory cytokine secretion and oxidative stress in HepG2 cells [16]. Current evidence also indicates an inverse association between a dairy-rich diet and obesity-associated inflammation. The dairy-supplemented diet resulted in significantly lower inflammatory markers, whereas soy exerted no significant effect [17]. Furthermore, oral administration of milk casein-derived tripeptide Val-Pro-Pro exerted anti-inflammatory effects on the adipose tissue of high-fat diet fed mice [18]. Casein glycomacropeptide (GMP), which is a casein-derived whey peptide, deserves much interest for its proposed biological activities, such as inhibiting intestinal bacterial adhesion and inhibition from binding of cholera toxin (CT) to Chinese hamster ovary (CHO)-K1 cells [19,20]. Our previous studies have demonstrated that GMP supplementation efficiently suppresses the proliferation, differentiation and lipid accumulation of *in vitro* pre-adipocytes [21]. GMP can also reduce plasma total cholesterol and slightly ameliorate the inflammatory state in obese rats [22]. In addition, the growth-promoting activity of GMP hydrolysates on *Lactobacillus bulgaricus* and *Streptococcus thermophilus* was higher than that of native GMP [23]. Whether the inhibitory activity of GMP on inflammation can be improved through enzymatic modification remains to be clarified.

Collectively, these data suggest that low-grade inflammation is an early instigator of metabolic syndrome and that the inflammatory state of macrophages is a potentially useful therapeutic target for inflammation-related disorders. Therefore, the present study aims to prepare enzymatic hydrolysates from GMP under different enzymatic conditions and to determine the inhibitory effects and underlying mechanism of GMP-derived peptides on the inflammatory response in LPS-stimulated RAW264.7 macrophages. Whether GMP-derived peptides may neutralize the inflammatory potential of LPS by binding directly to LPS or by preventing the interaction of LPS with LPS receptors at the macrophage surface membrane was also investigated.

## 2. Experimental Section 

### 2.1. Reagents and Materials 

Casein glycomacropeptide (GMP, the GMP content of protein is a minimum of 95%) was provided by Arla Co. (Sønderhøj, Denmark). Lipopolysaccharides (LPS) from *E. coli* 011:B4, fluorescein isothiocyanate-lipopolysaccharides (FITC-LPS) from *E. coli* 011:B4, papain (EC 3.4.22.21, with an activity of 2 units per milligram), pepsin (EC 3.4.23.1, with an activity of 3,000 units per milligram) and methylthiazolyldiphenyl-tetrazolium bromide (MTT) were purchased from Sigma-Aldrich (St. Louis, MO, USA). Alcalase (EC 3.4.21.62, with an activity of 2.4 Anson units per gram; endoproteinase from *Bacillus licheniformis*) was provided by Novo Nordisk Biochem. Inc. (Franklinton, NC, USA). All antibodies used in this investigation were obtained from commercial sources. Mouse monoclonal inhibitor of κB-α (IκBα, No. 4814) antibody and rabbit monoclonal Nuclear factor-κB p65 subunit (NF-κB p65, No. 8242), phosphorylated inhibitor of κB-α (pIκBα, No. 2859), IκB kinase-α (IKKα, No. 2682), IKKβ (No. 2370) and phosphorylated IKKα/β (pIKKα/β, No. 2697) antibodies were purchased from Cell Signaling Technology (CST, Beverly, MA, USA). β-actin, histone H4 and horseradish peroxidase-conjugated anti-species (mouse and rabbit) secondary antibodies were purchased from Beyotime Biotech (Haimen, Jiangsu, China). 

### 2.2. Preparation of GMP Hydrolysates

GMP was dissolved in distilled water at a concentration of 5% (w/v). The protein solution was adjusted to optimal pH using 1 N NaOH or 1 N HCl. Afterward, the protein solution was hydrolysed by alcalase, pepsin and papain under their optimal temperatures, respectively. During the hydrolysis process, 1 N NaOH solution was continuously added to maintain the optimal pH of the reaction system. After different hydrolysis periods (0, 0.25, 0.5, 1, 2, 3, 4, 5, 6 and 7 h), samples were collected and immediately heated in a water bath at 85 °C for 20 min to inactivate protease and stop the hydrolysis reaction. When cooled to room temperature, the hydrolysate solutions were centrifuged at 4000× *g* for 20 min to remove unhydrolyzed protein. The supernatant was collected and lyophilized.

### 2.3. Cell Viability Analysis

Murine macrophage-like RAW264.7 cell line was obtained from American Type Culture Collection (Manassas, VA, USA). Cells were cultured at 37 °C with 5% carbon dioxide (CO_2_) in Dulbecco’s Modified Eagle’s Medium (DMEM, HyClone, Logan, UT, USA) containing 10% fetal bovine serum (FBS, Bioin, Israel), 100 U mL^−1^ penicillin and 100 μg mL^−1^ streptomycin (Invitrogen, Carlsbad, CA, USA). Cell viability was measured by the MTT assay [24]. Briefly, RAW264.7 macrophages were cultured with the tested sample at a range of concentrations (0.25, 0.5, 1.0 and 2.0 mg mL^−1^) for 48 h. After incubation, 20 μL of MTT stock solution (5 mg mL^−1^) were added to each well. After incubation for 4 h, the reaction was terminated, and the formed formazan was solubilized by the addition of 100% dimethyl sulfoxide (DMSO). Finally, the absorbance was monitored by a microplate reader (Bio-Rad, Model 680, Hercules, CA, USA) at a wavelength of 570 nm. 

### 2.4. Measurement of NO Production

RAW264.7 macrophages were seeded onto 96-well plates at a concentration of 2 × 10^4^ cells well^−1^ and allowed to adhere overnight. The cells were preincubated with tested samples or PBS (pH 7.4) for 12 h at 37 °C prior to LPS (1 μg mL^−1^) treatment for 24 h. After incubation, the cell-free culture medium was collected. The nitrite accumulated in culture medium was measured as an indicator of NO production in macrophages, based on the Griess reaction system [25]. Briefly, 100 μL of cell culture medium were mixed with an equal volume of Griess reagent (Sigma, USA). After being incubated at room temperature for 10 min, the absorbance was measured at 540 nm. The concentrations of nitrite present in the samples were calculated from a standard curve established with serial dilutions of NaNO_2_ in culture medium.

### 2.5. RNA Isolation and Reverse Transcriptase-Polymerase Chain Reaction

RAW264.7 macrophages (1 × 10^6^ cells) were preincubated with tested samples or PBS for 12 h at 37 °C following LPS (1 μg mL^−1^) stimulation for 24 h. Total cellular RNA was isolated using TRIzol reagent (Tiangen Biotech, Beijing, China) according to the manufacturer’s instructions. The total RNA (3 μg) was converted to cDNA using the TIANScript RT Kit (Tiangen, China). PCR assay was performed at an annealing temperature of 60 °C with 35 amplification cycles for glyceraldehyde-3-phosphate dehydrogenase (GAPDH), TNF-α, IL-1β, iNOS, myeloid differentiation primary response gene 88 (MyD88) and toll-like receptor 4 (TLR4) using specific primers (as shown in Section 2.6). The amplified PCR products were separated with 1.2% agarose gel in Tris-acetic acid-EDTA (TAE) buffer and visualized by ethidium bromide (EB) staining. The GAPDH gene was used as an internal standard to normalize the amount of total RNA present in each reaction.

### 2.6. Real-Time Quantitative Polymerase Chain Reaction

Quantitative RT-PCR was carried out with a Techne Quantica real-time PCR detection system (Techne, Staffordshire, UK) using SYBR^®^ Premix Ex Taq™ (Takara, Otsu, Shiga, Japan) in a 20-μL reaction volume. The following primers were used to amplify target cDNA: sense strand TNF-α, 5′-CATCTTCTCAAAATTCGAGTGACAA-3′, and anti-sense strand TNF-α, 5′-TGGGAGTAGACAAGGTACAACCC-3′; sense strand IL-1β, 5′-TCAAATCTCGCAGCAGCACATC-3′, and anti-sense strand IL-1β, 5′-CCAGCAGGTTATCATCATCATCCC-3′; sense strand iNOS, 5′-GTTCTCAGCCCAACAATACAAGA-3′, and anti-sense strand iNOS, 5′-GTGGACGGGTCGATGTCAC-3′; sense strand TLR4, 5′-CTTCATTCAAGACCAAGCCTTTC-3′, and anti-sense strand TLR4, 5′-AACCGATGGACGTGTAAACCAG-3′; sense strand MyD88, 5′-CACTCGCAGTTTGTTGGATG-3′, and anti-sense strand MyD88, 5′-CGCAGGATACTGGGAAAGTC-3′; sense strand GAPDH, 5′-TGGCAAAGTGGAGATTGTTGC-3′, and anti-sense strand GAPDH, 5′-AAGATGGTGATGGGCTTCCCG-3′. Real-time quantitative PCR was performed starting with a denaturation at 95 °C for 120 s followed by 40 cycles of 95 °C for 5 s, 60 °C for 30 s and 72 °C for 30 s. The SYBR green fluorescence was read at the end of each extension step (72 °C). The ΔΔ Ct method was used to obtain fold-changes in mRNA abundance [26]. Data were normalized by the level of GAPDH mRNA expression in each sample and presented as the fold change relative to the control.

### 2.7. Determination of LPS-Binding Activity (TAL Assay)

LPS-binding ability was assessed using a quantitative chromogenic Tachypleus amebocyte lysate (TAL) assay kit (Xiamen, Fujian Province, China). Experiments were carried out in accordance with the institutional guidelines. Briefly, LPS (1 μg mL^−1^) was incubated with various concentrations of tested sample (0.0625, 0.125, 0.25, 0.5, 1.0 and 2.0 mg mL^−1^) at 37 °C for 30 min. Then, 100 μL of LPS standard solution or LPS mixed with tested sample were added in triplicate to 100 μL of TAL in a pyrogen-free tube. After 10 min of incubation at 37 °C, the TAL-sample mixture was incubated with a pre-warmed substrate solution at 37 °C for an additional 6 min. The absorbance of the TAL-sample mixture was measured at 545 nm by a spectrophotometer (Unico UV-2600, Shanghai, China). The inhibition of substrate color production as a function of inhibition of TAL enzyme activity was estimated, and the percentage of LPS binding was calculated.

### 2.8. Flow Cytometry Analysis of LPS-Binding Assay

RAW264.7 macrophages were incubated with FITC-conjugated LPS (1 μg mL^−1^) in the absence or presence of various concentrations of tested sample (0.25, 0.5, 1.0 and 2.0 mg mL^−1^; diluted in DMEM containing 10% FBS) for 15 min at 37 °C. After incubation, the cells were washed three times with PBS to remove unbound LPS and then fixed in 2% paraformaldehyde. The binding of FITC-conjugated LPS was analyzed on a FACSCalibur flow cytometer (BD Biosciences, San Jose, CA, USA). Data were analyzed by Flowjo 7.6.1 software (Tree Star, USA) and expressed as median fluorescence intensity.

### 2.9. Flow Cytometry Analysis of Toll-Like Receptor 4 Expression on the Surface of RAW264.7 Macrophages 

RAW264.7 macrophages (1 × 10^6^ cells) were pre-incubated with tested samples or PBS for 12 h at 37 °C following LPS stimulation (1 μg mL^−1^) for 24 h. After stimulation with LPS, the cells were washed three times with PBS and then harvested with 0.25% trypsin (Gibco, Carlsbad, CA, USA). TLR4 protein on the cell surface was analyzed by fluorescence activated cell sorting (FACS) with anti-TLR4-PE by a slight modification of previously described methods [27]. Briefly, the cells were washed three times and resuspended in FACS buffer (PBS containing 1% BSA). Afterwards, the cells were stained with anti-mouse CD284 (TLR4) PE or mouse IgG1 k isotype control PE (eBioscience, St Diego, CA, USA) diluted in FACS buffer at a final concentration of 0.25 μg per tube for 60 min at 4 °C in the dark. After incubation with antibody/isotype control, the cells were washed twice with ice-cold PBS and subjected to flow cytometric analysis. Results are expressed as the fold change relative to the control.

### 2.10. Western Blot Analysis

RAW264.7 macrophages (2 × 10^6^ cells mL^−1^) were pretreated with tested samples or PBS for 12 h in 100-mm cell culture dishes (Corning, Tewksbury, MA, USA) following stimulation with LPS (1 μg mL^−1^) for 30 min. The cells were harvested with cell lysis buffer (50 mM Tris-HCl, pH 7.4, 150 mM NaCl, 5 mM EDTA, 50 mM NaF, 1% Triton X-100, 1 mM sodium orthovanadate, 1 mM phenyl methane sulfonyl fluoride, 1 mg mL^−1^ aprotinin, 2 μg mL^−1^ pepstatin A and 2 μg mL^−1^ leupeptin; Beyotime, Haimen, Jiangsu, China) containing 1 mM phenylmethylsulfonyl fluoride (PMSF, Sigma-Aldrich). After centrifugation at 6800× *g* for 15 min at 4 °C, the protein was quantified using the bicinchoninic acid assay (BCA assay). Nuclear and cytoplasmic protein samples were prepared using the nuclear and cytoplasmic extraction reagent kit (Beyotime, China) for the NF-κB translocation assay. Equal amounts of protein samples (20 μg) were subjected to 10% sodium dodecyl sulfate-polyacrylamide gel electrophoresis (SDS-PAGE) and subsequently transferred to polyvinylidene difluoride (PVDF) membranes (Millipore, Billerica, MA, USA). These membranes were blocked with 5% skim milk in PBS-T solution (0.05% Tween-20 in 1× PBS solution) at room temperature for 2 h and subsequently incubated overnight with the appropriate primary antibody at 4 °C. After washing with PBS-T solution, the PVDF membranes were incubated with peroxidase conjugated secondary antibody for 1 h. Immunolabeled proteins were detected using an Immobilon Western chemiluminescent HRP substrate (ECL, Millipore).

### 2.11. Statistical Analysis

The data are expressed as the means ± standard deviations (SD). The differences among the groups were analyzed by one-way analysis variance (ANOVA) followed by Tukey’s method. A *p*-value less than 0.05 was considered statistically significant.

## 3. Results 

### 3.1. Effect of GMP Hydrolysates on NO Production in LPS-Stimulated RAW264.7 Macrophages 

**Figure 1:**
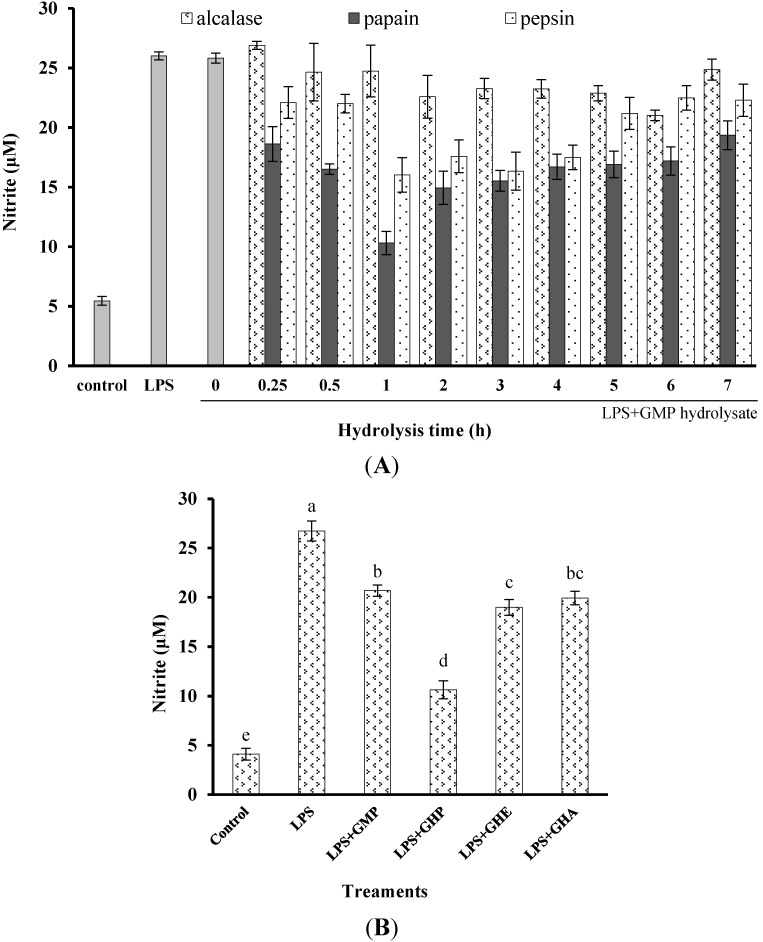

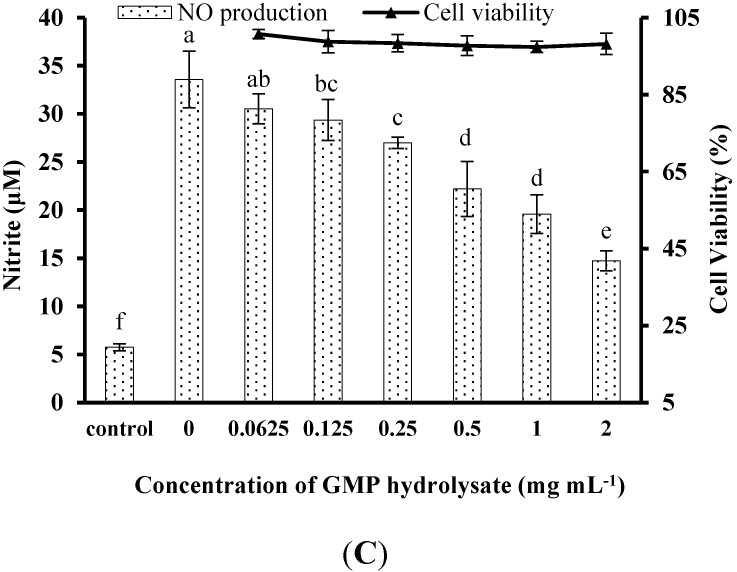
Inhibitory activity of casein glycomacropeptide (GMP) and its hydrolysates on NO production in LPS-stimulated RAW264.7 macrophages. (**A**) Effect of GMP hydrolysates obtained with alcalase, pepsin and papain at various hydrolysis periods on NO production in LPS-stimulated RAW264.7 macrophages; (**B**) Effect of different GMP hydrolysates on NO production in LPS-stimulated RAW264.7 macrophages. GMP hydrolysates produced by alcalase at 6 h hydrolysis, pepsin at 1 h hydrolysis and papain at 1 h hydrolysis were referred to as GHA, GHE and GHP, respectively. (**C**) LPS-stimulated NO production in RAW264.7 macrophages was suppressed by GHP in a dose-dependent manner. The results are presented as the means ± SD of four independent experiments. Means with different letters are significantly different from each other at *p* < 0.05.

To determine and compare the inhibitory activity of GMP hydrolysates on inflammatory responses in LPS-stimulated RAW264.7 macrophages, the effects of GMP hydrolysates prepared by alcalase, pepsin and papain for different periods on LPS-stimulated NO production were measured (Figure 1A). After treatment with LPS for 24 h, the NO concentration in the medium markedly increased five-fold to 27.33 ± 1.36 μM in comparison with the control group. Compared with native GMP (without hydrolysis), GMP hydrolysates obtained at the optimum hydrolysis period markedly decreased LPS-stimulated NO production. The optimal hydrolysis time for GMP by alcalase, pepsin and papain was 6 h, 1 h and 1 h, respectively. GMP hydrolysate produced by papain at 1 h hydrolysis (GHP) was the best inhibitor of LPS-stimulated NO production in RAW264.7 cells, which was significantly better than that of GMP hydrolysates prepared with alcalase (GHA) and pepsin (GHE) at the optimum hydrolysis period (*p* < 0.05), as shown in Figure 1B. Considering the highest inhibitory activity of LPS-mediated NO production in RAW264.7 macrophages (decreased to 10.63 ± 1.36 μM), GHP was used for further study. 

The obtained GHP significantly suppressed NO production in LPS-stimulated RAW264.7 macrophages in a dose-dependent manner at a concentration range from 0.125 mg mL^−1^ to 2.0 mg mL^−1^, as shown in Figure 1C. The NO production induced by LPS (1 μg mL^−1^) was markedly reduced by 60.21% in the presence of GHP at a concentration of 2.0 mg mL^−1^. To exclude the possibility that the decrease in NO production by GHP was due to cell growth inhibition, the effect of GHP on cell viability was determined by the MTT assay. Since GHP did not exhibit any noticeable cytotoxicity at experimental concentrations (Figure 1C), the possibility that the NO production was inhibited by the cytotoxicity of GHP could be excluded.

### 3.2. Effect of GHP on Inflammatory Gene Expression in LPS-Stimulated RAW264.7 Macrophages

**Figure 2:**
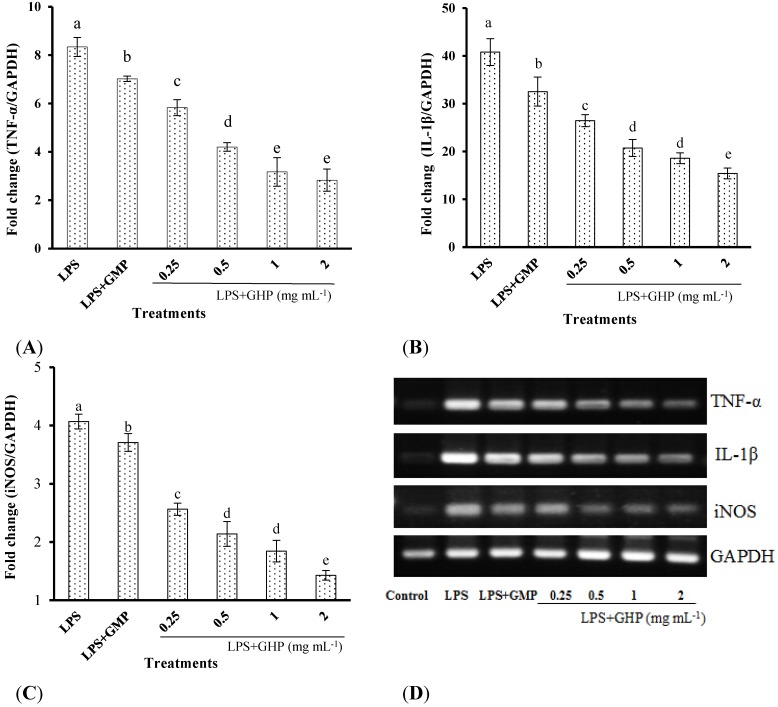
Inhibitory effects of the GMP hydrolysate obtained with papain for 1 h hydrolysis (GHP) on tumor necrosis factor (TNF)-α (**A**), interleukin (IL)-1β (**B**) and inducible nitric oxide synthase (iNOS) (**C**) mRNA expression in LPS-stimulated RAW264.7 macrophages. Results (means ± SD of three independent experiments) are expressed as the mean fold change relative to the control (untreated) cells. Different letters within columns indicate significant difference (*p* < 0.05). (**D**) The TNF-α, IL-1β and iNOS mRNA levels were determined by RT-PCR.

The inhibitory effects of GHP on LPS-stimulated TNF-α, IL-1β and iNOS gene expression in RAW264.7 macrophages were determined by RT-qPCR. LPS treatment significantly increased TNF-α (Figure 2A), IL-1β (Figure 2B) and iNOS (Figure 2C) mRNA expression (mRNA fold changes of 8.34 ± 0.40, 40.79 ± 2.82 and 4.07 ± 0.13, respectively). Pretreatment of RAW264.7 macrophages with different concentrations of GHP produced a significant dose-dependent decrease in TNF-α, IL-1β and iNOS mRNA expression induced by LPS (*p* < 0.05). TNF-α, IL-1β and iNOS mRNA expression was significantly decreased by 66.16%, 62.22% and 64.89%, respectively, in the presence of GHP at a concentration of 2.0 mg mL^−1^. The gel images of RT-PCR are shown in Figure 2D.

### 3.3. In Vitro LPS-Binding Activity of GHP

**Figure 3:**
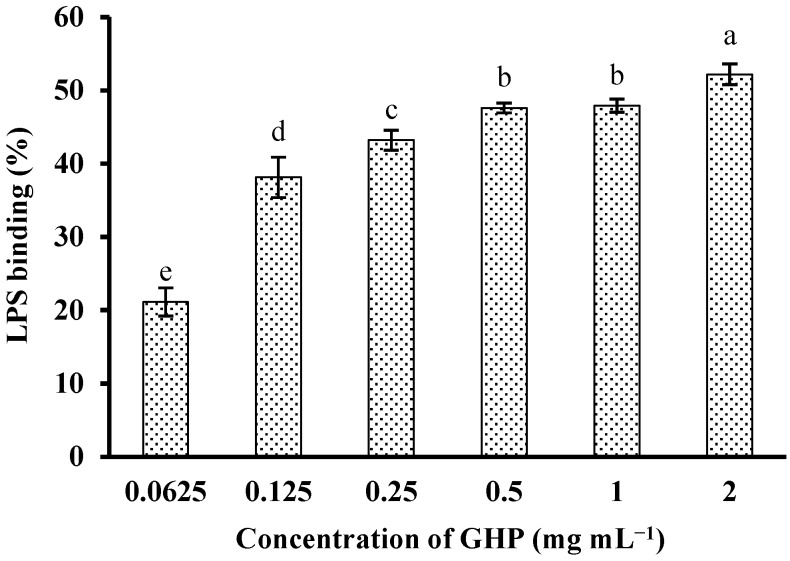
*In vitro* LPS-binding activity of GMP hydrolysate obtained with papain for 1 h hydrolysis (GHP). LPS (1 μg mL^−1^) was incubated with various concentrations of GHP (0.0625–2.0 mg mL^−1^) and then was detected by the Tachypleus amebocyte lysate (TAL) assay. The results are presented as the means ± SD of the data from three independent experiments. Columns that do not share the same letter are significantly different from each other at *p* < 0.05.

In an attempt to investigate whether GHP could block the biological effects of LPS, binding of GHP to LPS was determined by measuring its efficacy to inhibit the LPS-induced activation of the TAL enzyme. GHP showed significant binding to LPS in a concentration-dependent manner (*p* < 0.05), as shown in Figure 3. GHP at a concentration of 2.0 mg mL^−1^ exhibited 52.19% ± 1.43% inhibition of the TAL enzyme as a consequence of higher LPS binding efficiency. The result indicated that GHP could attenuate LPS-stimulated inflammatory response in RAW264.7 macrophages, probably due to its ability to bind LPS.

### 3.4. Effect of GHP on the Binding of FITC-LPS to the Cell Surface of RAW264.7 Macrophages 

**Figure 4:**
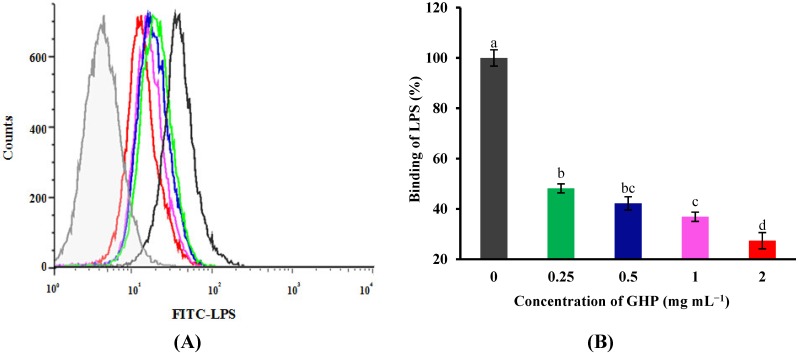
Flow cytometric analysis of the binding of fluorescein isothiocyanate (FITC)-labeled LPS to the surface of RAW264.7 macrophages in the absence or presence of GMP hydrolysate obtained with papain for 1 h hydrolysis (GHP). Cells were incubated with 1 μg mL^−1^ FITC-LPS and different concentrations of GHP (0.25–2.0 mg mL^−1^). (**A**) Background fluorescence is shown as a gray profile. The binding of LPS to the cell surface was analyzed by FACS. (**B**) The FITC-LPS binding to RAW264.7 macrophages was expressed as the percentage of that with FITC-LPS alone. The values for macrophages that had bound FITC-LPS alone (red line) were set as 100%. The results are presented as the means ± SD of the data from three independent experiments. Columns that do not share the same letter are significantly different from each other at *p* < 0.05.

To further analyze whether GHP can inhibit the direct binding of LPS to the RAW264.7 macrophage surface, flow cytometric assay was used to examine the effect of pre-incubation of macrophages with GHP on FITC-LPS binding, and untreated cells served as controls. FITC-LPS obviously bound to the macrophages surface compared with the control group, as shown in Figure 4. However, the binding of FITC-LPS to RAW264.7 macrophages was blocked by pretreatment with GHP in a dose-dependent manner. GHP inhibited the binding of FITC-LPS to the surface of RAW264.7 macrophages by 72.62% at a concentration of 2 mg mL^−1^. The result suggested that GHP could attenuate LPS-stimulated inflammatory response in RAW264.7 macrophages, probably due to the suppression of LPS binding to the cell surface.

### 3.5. GHP Reduced LPS-Stimulated Inflammatory Response in RAW264.7 Macrophages through the TLR4/MyD88 Signaling Pathway 

**Figure 5:**
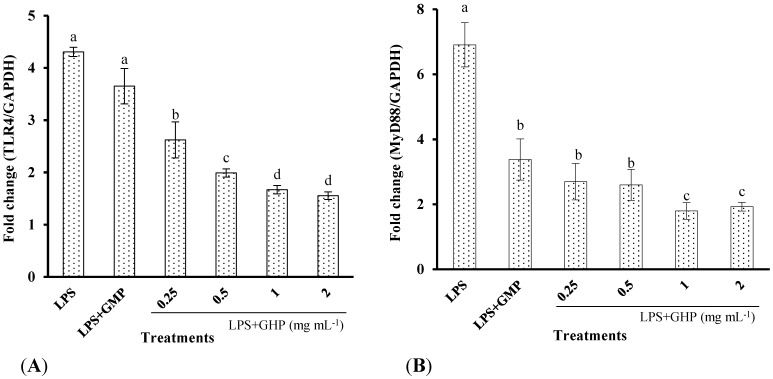

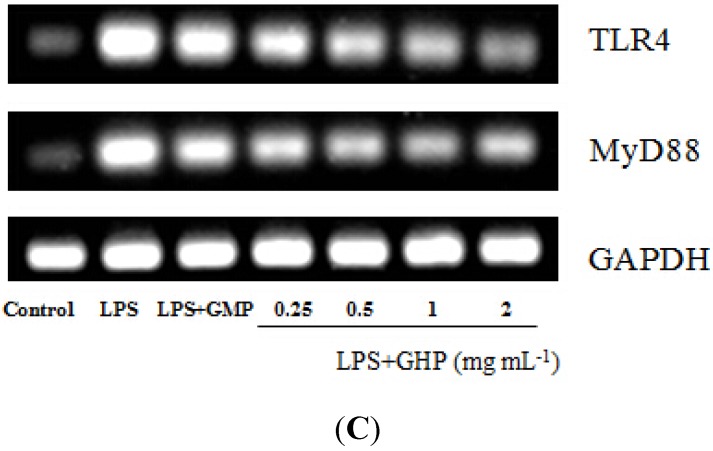
Effects of GMP hydrolysate obtained with papain for 1 h hydrolysis (GHP) on toll-like receptor 4 (TLR4) and myeloid differentiation primary response gene 88 (MyD88) mRNA expression in LPS-stimulated RAW264.7 macrophages. (**A**) TLR4 mRNA expression is shown as the means ± SD of the relative fold increase compared to the control (untreated) cells; (**B**) MyD88 mRNA expression is shown as the means ± SD of the relative fold increase compared to the control (untreated) cells. Columns that do not share the same letter are significantly different from each other at *p* < 0.05. (**C**) The TLR4 and MyD88 mRNA levels were determined by RT-PCR.

**Figure 6:**
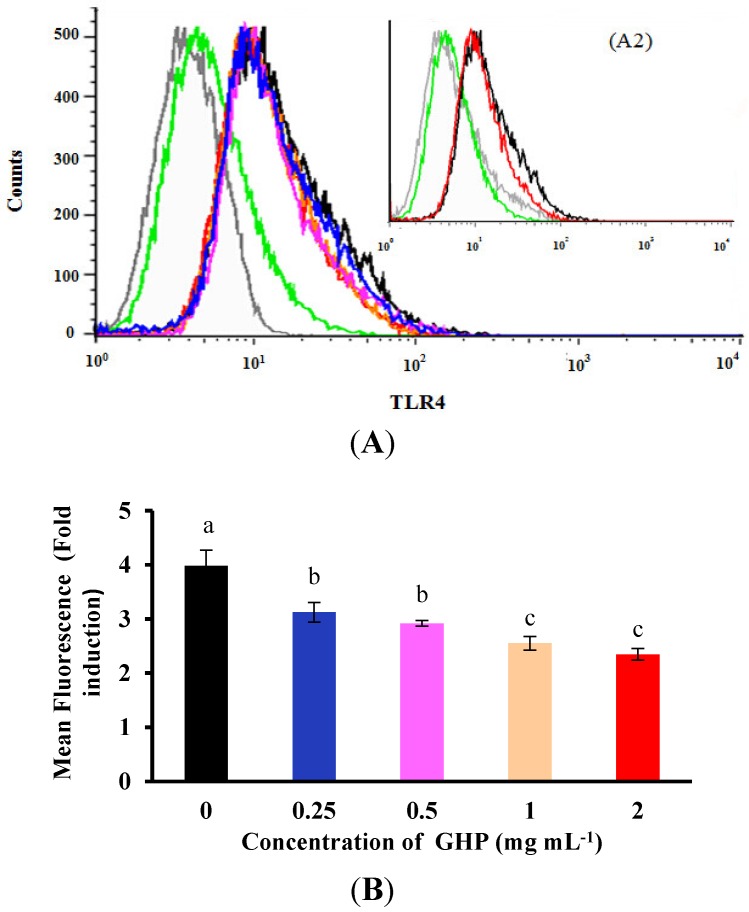
Effects of GMP hydrolysate obtained with papain for 1 h hydrolysis (GHP) on toll-like receptor 4 (TLR4) protein expression in LPS-stimulated RAW264.7 macrophages. (**A1**) Representative flow cytometry histogram data. RAW264.7 macrophages were pretreated with different concentrations of GHP (0.25–2.0 mg mL^−1^) and treated with LPS (1 μg mL^−1^). TLR4-positive cells treated with LPS (black line), untreated cells (green line) and isotype-matched control for TLR4 staining (grey line). (**A2**) Representative flow cytometry histogram data. RAW264.7 macrophages were pretreated with 2.0 mg mL^−1^ GHP and treated with LPS (1 μg mL^−1^). TLR4-positive cells treated with LPS (black line) and pretreatment of GHP to LPS-stimulated RAW264.7 macrophages (red line). (**B**) The results from fluorescence activated cell sorting (FACS) analysis are expressed as the mean fluorescence intensity (MFI) fold induction, calculated by dividing the MFI values of stimulated cells with the values of control cells. The results are presented as the means ± SD of the data from three independent experiments. Columns that do not share the same letter are significantly different from each other at *p* < 0.05.

To assess the inhibitory effects of GHP on the LPS-induced TLR4 activation, the TLR4 surface protein level in RAW264.7 macrophages was determined by flow cytometry using an anti-TLR4-PE. As shown in Figure 5 and Figure 6, TLR4 mRNA expression and protein level increased 4.31 ± 0.09-fold and 3.98 ± 0.29-fold after LPS stimulation, respectively. In contrast, pretreatment of the cells with GHP exhibited a significant inhibition on the increased expression of TLR4 in a dose-dependent manner (*p* < 0.05). MyD88 is an important signaling adaptor in TLR4 signaling. After LPS treatment, the MyD88 mRNA expression was markedly increased. GHP pretreatment significantly downregulated the MyD88 mRNA expression (*p* < 0.05).

### 3.6. Effect of GHP on NF-κB Activation in LPS-Stimulated RAW264.7 Macrophages

**Figure 7:**
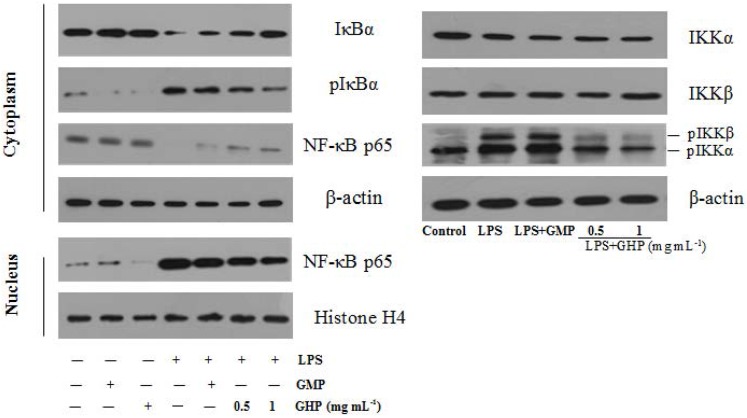
Effects of GMP hydrolysate obtained with papain for 1 h hydrolysis (GHP) on LPS-induced Nuclear factor-κB (NF-κB) activation in RAW264.7 macrophages. RAW264.7 macrophages were pretreated with/without the indicated concentrations of GHP and then stimulated with LPS (1 μg mL^−1^) for 30 min. Cell lysates were analyzed by Western blotting using specific antibodies against inhibitor of κB-α (IκBα), phosphorylated inhibitor of κB-α (pIκBα), Nuclear factor-κB p65 subunit (NF-κB p65) and phosphorylated IκB kinase-α/β (pIKKα/β).

To further explore the underlying mechanism of GHP on the inflammatory responses in LPS-stimulated RAW264.7 macrophages, the protein levels of NF-κB p65, IκBα, pIκBα and pIKKα/β were confirmed by Western blot analysis, as shown in Figure 7. NF-κB p65 protein was less expressed in the cytosol fraction and strongly expressed in the nuclear fraction after stimulation with 1.0 μg mL^−1^ LPS. However, compared with the LPS-only group, GHP treatment at a concentration of 1.0 mg mL^−1^ significantly blocked p65 nuclear translocation. Furthermore, treatment with LPS alone increased the phosphorylation and degradation of IκBα in RAW264.7 macrophages. GHP treatment was shown to inhibit LPS-induced phosphorylation and degradation of IκBα. Activation of the IKK complex by LPS induces phosphorylation and degradation of IκBα, leading to the nuclear translocation of NF-κB. LPS was found to strongly induce IKKα/β phosphorylation, whereas GHP markedly inhibited this phosphorylation.

## 4. Discussion 

Metabolic diseases are commonly associated with systemic evidence of inflammation and development of insulin resistance. An increase in plasma LPS concentration combined with macrophages activation in response to inflammatory stimuli may contribute directly to insulin resistance through the production of inflammatory mediators. TNF-α and IL-1β could affect insulin signaling independent of insulin receptor substrate (IRS), leading to a decrease in insulin-stimulated glucose uptake [28,29]. The iNOS-derived NO was required for the development of sepsis-induced skeletal muscle insulin resistance [30]. Therefore, it is hypothesized that targeting chronic LPS infusion and related systematic inflammation may provide a therapeutic route for metabolic disorder treatment. Several naturally occurring proteins, such as adiponectin and lactoferrin, possess LPS-binding capacity, resulting in the reduction of the inflammatory response and alleviation of metabolic disorders [31,32]. Our data indicate that the inhibitory activity of GMP hydrolysate produced by papain at a 1-h hydrolysis time (GHP) on LPS-stimulated inflammatory response in RAW264.7 macrophages was higher than that of native GMP. GHP significantly inhibited iNOS mRNA expression and subsequent NO synthesis in LPS-stimulated RAW264.7 macrophages. In addition, LPS-stimulated mRNA expression of TNF-α and IL-1β was also inhibited by pretreatment with GHP in a dose-dependent manner. Moreover, it has been determined that GHP possesses LPS-binding activity under the TAL assay. LPS-binding protein derived from Limulus can bind to lipid A, which is responsible for the endotoxic activity of LPS *in vitro* [33,34]. GHP may form a complex with lipid A of LPS and, thus, partially inhibit the LPS-induced release of pro-inflammatory mediators by macrophages. These findings suggest that because of its LPS-binding activity and inhibitory effects on LPS-stimulated inflammatory response in macrophages, GHP may have therapeutic potential in the treatment of macrophage-related inflammatory disorders.

Termination of the inflammatory response by blocking multiple pathways of the inflammation-signaling process is vital to maintain overall health. LPS interacts with toll-like receptor (TLR)-4 through binding to the co-receptor myeloid differentiation protein-2 (MD-2), resulting in downstream inflammatory events, which may be responsible for the development of metabolic diseases [35,36]. Previous study has shown that chitosan oligosaccharides significantly inhibited binding of LPS to the TLR4/MD-2 receptor complex, thus attenuating the production of pro-inflammatory mediators [37]. With a bone marrow transfer system, TLR4-deficient macrophages were shown to protect against diet-induced insulin resistance [38]. Moreover, the adaptor molecule myeloid differentiation factor 88 (MyD88) associates with the cytoplasmic domains of TLR4 to organize the initiation of a signaling cascade. Macrophages from MyD88^−/−^ mice are defective in many TLR4-mediated responses, such as LPS-induced secretion of inflammatory mediators [39]. Activation of NF-κB occurs downstream of TLR4/MyD88 and plays critical roles in the low-grade chronic inflammatory state. Long-term *in vivo* imaging of NF-κB activation identified adipose tissue macrophages (ATMs) as the major sites of NF-κB induction with diet-induced obesity [40]. In the present study, our data demonstrated that increased expression of TLR4 and Myd88 was concentration-dependently reduced in the presence of GHP. In addition, pretreatment with GHP markedly prevented the interaction of FITC-LPS with RAW264.7 macrophages to interrupt LPS engagement with the TLR4/MD2 receptor complex. NF-κB is dissociated from IκB and is translocated to the nucleus for promoting target gene expression [41]. The inhibitory effects of GHP on inflammatory gene expression in RAW264.7 cells appeared to involve inhibition of LPS-stimulated NF-κB activation by blocking IKK-induced IκB phosphorylation and translocation of the NF-κB/p65 protein to the nucleus. Our results have shown that GHP may prevent LPS-stimulated inflammatory response through negative regulation of the TLR4/MyD88/NF-κB pathway in RAW264.7 macrophages. Interestingly, a significant factor in the relationship between obesity and inflammation is the infiuence of the TLR4 signaling pathway. TLR4 was shown to be required not only for LPS-stimulated inflammatory response, but also for responses to nonbacterial ligands, such as free fatty acids (FFAs) [42]. Because of a pathophysiologic role for FFA-induced TLR4 activation in inflammation and insulin resistance, it is hypothesized that GHP, the inhibitor for bacterial LPS-induced inflammatory response, may contribute to the control of endogenous lipid-induced insulin resistance. The follow-up work on the suppression of FFAs-induced inflammatory changes in macrophages by GHP will be carried out in the future. 

## 5. Conclusions

The results obtained in the present study demonstrated that GHP showed a more significant inhibitory effect on LPS-stimulated inflammatory response than intact GMP. GHP was able to inhibit inflammation-related NO production and iNOS mRNA expression, as well as TNF-α and IL-1β mRNA expression in LPS-stimulated RAW264.7 macrophages. These effects were mediated, at least partially, by attenuating TLR4-mediated NF-κB activation in a MyD88-dependent pathway. In particular, our findings clearly showed that GHP could be a potential inhibitive effector of LPS, decreasing LPS-stimulated macrophage inflammatory reaction and signaling through significantly inhibiting the binding of LPS to the TLR4/MD-2 receptor complex. These results suggest that the obtained GHP may be a useful preventive and therapeutic agent against LPS infusion and LPS-related inflammation. As the study was done *in vitro*, further studies in animal models are needed to test this hypothesis.
